# Editorial: Islet cell development, heterogeneity and regeneration

**DOI:** 10.3389/fendo.2024.1404839

**Published:** 2024-05-17

**Authors:** Ansarullah, Adriana Migliorini, Mostafa Bakhti

**Affiliations:** ^1^ Center for Biometric Analysis, The Jackson Laboratory, Bar Harbor, ME, United States; ^2^ McEwen Stem Cell Institute, University Health Network, Toronto, ON, Canada; ^3^ Institute of Diabetes and Regeneration Research, Helmholtz Center Munich, Neuherberg, Germany; ^4^ German Center for Diabetes Research, Neuherberg, Germany

**Keywords:** beta cell, islet, diabetes, regeneration, cell replacement therapies

The loss or malfunction of insulin-producing β-cells leads to the onset of diabetes. Restoring β-cell function and mass from renewable sources has emerged as a promising therapeutic avenue (1, 2). However, significant hurdles still impede the clinical implementation, primarily stemming from gaps in our understanding of β-cell biology. Among the key challenges are the limited availability of functional stem cell-derived β (SC-β) cells, suboptimal transplantation techniques, the need to elucidate pathways underlying β-cell failure and develop interventions to preserve or restore their function, alongside with the need for novel tools to comprehensively study islet architecture and function. This Research Topic aims to assess the current state of knowledge regarding β-cell development and function, the mechanistic underpinnings of β-cell dysfunction, and potential strategies for restoring their identity and functionality. One original article explores a novel driver regulating endocrinogenesis, and two review articles discuss metabolic adaptation during SC-β cell generation and strategies for improved islet transplantation. Furthermore, three original articles investigate the roles of long non-coding RNAs (lncRNAs) in islet cell loss, the beneficial effects of a plant-derived compound on glucose homeostasis, and a novel liver-pancreas axis enhancing β-cell function. Finally, two original articles introduce tools and models for exploring islet architecture and β-cell function.

Compared to rodent studies, our understanding of the programs driving human β-cell development and regulating their function and survival remains limited (3, 4). Two articles have contributed to bridging such gaps. In one study, Cota et al. combined *in vitro* stem cell differentiation with CRISPR-Cas9 technology to explore the function of NEUROD2 during human endocrinogenesis. They found that this transcription factor is dispensable for endocrine cell induction and specification of SC-α and SC-β cells. However, whether NEUROD2 regulates the development of δ- and epsilon-cells warrants further investigation. In another study, Hossain et al., delved into the mechanisms dictating lncRNA-mediated post-transcriptional and epigenetic regulation in human β-cells. Through transplanting human islets into humanized mice, followed by the adoptive lymphocyte transfer using diabetic NOD mice, they induced human β-cell death and identified several lncRNA isoforms specific to and linked with function and survival of human β-cells. The potential of these identified lncRNAs as novel biomarkers for β-cell failure or therapeutic intervention still need to be further explored.

Despite notable progress, leveraging cell replacement therapy for diabetes requires better characterization of generated SC-β cells and refining SC-β/islet transplantation techniques (5, 6). In this context, Jasra et al. review the metabolic transformations during the reprogramming of somatic cells into induced pluripotent stem cells (iPSCs). They discuss the pivotal role of mitochondrial dynamics and the underlying mechanisms involved in iPSC reprogramming and their directed differentiation towards distinct cell types including SC-β cells. Additionally, the article touches on the metabolic adjustments required for iPSC differentiation towards SC-islets, which can aid in optimizing protocols for producing safe and functional SC-β cells. The other review article by Doherty et al. discusses the critical role of the native islet microenvironment, which undergoes disruption during the isolation process, eliciting tissue injury responses. It emphasizes the importance of reconstructing the islet niche by integrating non-endocrine components, such as vasculature, and extracellular matrix elements like the interstitial matrix and basement membrane. This approach holds promise not only for improving the success rate of human islet engraftment but also for advancing the bioengineering of SC-islets.

Different strategies have been undertaken to enhance the function of the dysfunctional β-cells (7). For instance, interest in plants and phytochemicals for managing blood glucose levels has surged, but the mechanisms behind their effectiveness are largely unexplored (8). In the work authored by Paul et al., the cellular mode of action of pheophorbide A, a derivative of chlorophyll, was investigated. They found that pheophorbide A influences the efficiency of glucose transporters (GLUTs), thus enhancing glucose uptake. The study proposes the GLUT1-Pheophorbide A complex as a promising therapeutic option for improving blood glucose levels in diabetes. In another study, Mahmoudi-Aznaveh et al., explored a novel perspective on liver-pancreas interplay in insulin-resistant conditions. They found that liver-derived exosomes enhance the expression of key β-cell markers such as insulin. This indicates that specific molecular cargoes within exosomes can regulate glucose homeostasis by enhancing β-cell functionality. Their findings emphasize the potential of exosomes as delivery systems for diabetes therapy, underscoring the need for further investigation into their molecular contents and impact on the functional response of β-cells in obesity and diabetes.

Advancing our understanding of β-cell biology requires the development of novel tools to probe into structure and function of islets. For instance, mathematical models can assist in elucidating the intricate interplay between metabolic signaling pathways and glucose homeostasis (9). Along this line, Maheshvare et al., employed the Systems Biology Markup Language (SBML) to construct a comprehensive kinetic model of glucose-stimulated insulin secretion (GSIS). By applying this model to both clinical and experimental datasets across different species, they were able to elucidate the associated alterations in glycolysis within pancreatic β-cells. In another advancement, Pfeifer et al. designed an open-source tool called “PyCreas” for fast and accurate quantitative analysis of islet architecture. To assess the efficacy of the PyCreas tool in quantifying cell distribution within islets across various metabolic states, they investigated the localization and distribution patterns of endocrine cells during gestation and prediabetes states in mouse models. This novel tool will allow a systematic quantitative approach for analyzing islet cell distribution that can aid in examining the pathophysiology of metabolic disorders.

The studies presented in this Research Topic enhance our understanding of β-cell biology, offering new avenues to explore islet structure, function, and β-cell regeneration. Moving forward, it is imperative to direct our focus toward leveraging the complexities of human β-cell development and physiology for improved cell therapy, and to explore diverse approaches for restoring β-cell mass or function *in vivo*. Additionally, understanding how different regenerative strategies can be tailored to suit the diverse needs of patients with diabetes complications is crucial and warrants further exploration. We commend the authors for their invaluable contributions to this Research Topic on islet biology.

## Author contributions

A: Writing – original draft, Writing – review & editing. AM: Writing – review & editing. MB: Writing – original draft, Writing – review & editing.
